# Large-scale inference of protein tissue origin in gram-positive sepsis plasma using quantitative targeted proteomics

**DOI:** 10.1038/ncomms10261

**Published:** 2016-01-06

**Authors:** Erik Malmström, Ola Kilsgård, Simon Hauri, Emanuel Smeds, Heiko Herwald, Lars Malmström, Johan Malmström

**Affiliations:** 1 Department of Clinical Sciences, Lund, Division of Infection Medicine, Lund University, BMC, Tornavägen 10, SE-22184 Lund, Sweden; 2 S3IT, University of Zurich, Winterthurerstrasse 190, CH-8057 Zurich, Switzerland

## Abstract

The plasma proteome is highly dynamic and variable, composed of proteins derived from surrounding tissues and cells. To investigate the complex processes that control the composition of the plasma proteome, we developed a mass spectrometry-based proteomics strategy to infer the origin of proteins detected in murine plasma. The strategy relies on the construction of a comprehensive protein tissue atlas from cells and highly vascularized organs using shotgun mass spectrometry. The protein tissue atlas was transformed to a spectral library for highly reproducible quantification of tissue-specific proteins directly in plasma using SWATH-like data-independent mass spectrometry analysis. We show that the method can determine drastic changes of tissue-specific protein profiles in blood plasma from mouse animal models with sepsis. The strategy can be extended to several other species advancing our understanding of the complex processes that contribute to the plasma proteome dynamics.

## Abstract (web summary)

Sepsis can lead to multiple organ failure that could potentially be reflected by change in plasma protein abundance. Here the authors describe a proteomics strategy that allows the determination of plasma proteins tissue origin in a quantitative manner for use as biomarkers—illustrated in a mouse model of sepsis.

## Graphical abstract

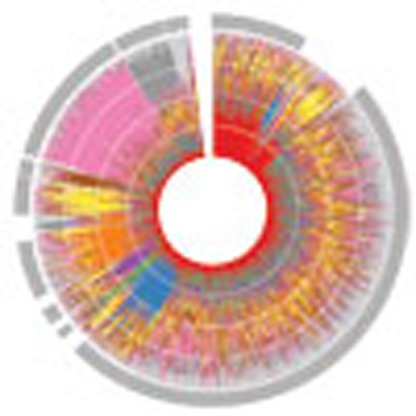

The blood plasma proteome is maintained by influx and efflux of proteins from surrounding cells and organs. The liver secretes the majority of the highly abundant plasma proteins, the so-called classical plasma proteins, involved in plasmas principal functions such as serving as transport medium, provide colloid osmotic pressure and maintaining hemostasis through the complement and coagulation systems. Blood plasma also contains numerous other tissue proteins that most likely do not contribute to the principal functions of blood plasma. This group of proteins exists in larger numbers^1^ than the classical plasma proteins and their role, if any, in the plasma is unclear. A subset of these proteins may be waste products resulting from the normal turnover of proteins and cells. It is probable that many, but not all, of the proteins found in plasma are under homeostatic control and play important or vital roles in healthy or diseased states.

Currently, detailed understanding of the factors behind the control of the blood plasma proteome is still missing. It remains unknown to what extent various tissues can alter the blood plasma composition under healthy and pathological conditions. The recent development of SWATH-like data-independent analysis mass spectrometry (DIA-MS) facilitates the acquisition of close-to-complete digital representations of analysed trypsin-cleaved proteomes from biological samples^2^. Importantly, protein identities and quantities are extracted from the DIA-MS maps using a spectral library constructed from previously acquired shotgun MS analyses^3^,^4^. Here we present how a priori constructed spectral libraries can be extended to include information regarding protein tissue distribution. On the basis of extensive shotgun MS analysis of several organs and cell types in mice, we created a tissue atlas, a distribution map of the tissue proteomes across highly vascularized organs and cells adjacent to the blood plasma. The resulting shotgun MS data were transformed to a spectral library, and based on the protein distribution map in the tissue atlas it is possible to infer protein origin of the detected proteins in the blood plasma.

We applied the spectral library and DIA-MS to characterize the blood plasma proteome dynamics during severe sepsis using mice as a model system. Sepsis is a life-threatening condition responsible for over 8 million deaths every year^5^. The pathogenesis of sepsis involves both exaggerated inflammation and immune suppression, caused by a dysregulated and overwhelming immune response to microbial pathogens present in the blood stream^6^,^7^. Sepsis is a multifactorial and systemic disease with rapid disease progression that can lead to vascular leakage and multiple organ failure. Thus, it can be expected that the complex pathogenesis in sepsis results in changes in tissue protein concentrations in the blood plasma. Here we show that DIA-MS analysis of septic blood plasma reveals a drastic reorganization of the blood plasma proteome related to disease severity. The plasma proteome response is complex and involves subgroups of proteins that are regulated differently. Part of the plasma proteome followed a dose-dependent reorganization, while another group of tissue proteins were increased in the most severely ill animals. The increase of tissue proteins in critically ill animals may indicate early signs of organ failure, a hallmark of sepsis pathology.

## Results

### Construction of a murine protein tissue atlas

**Figure 1:**
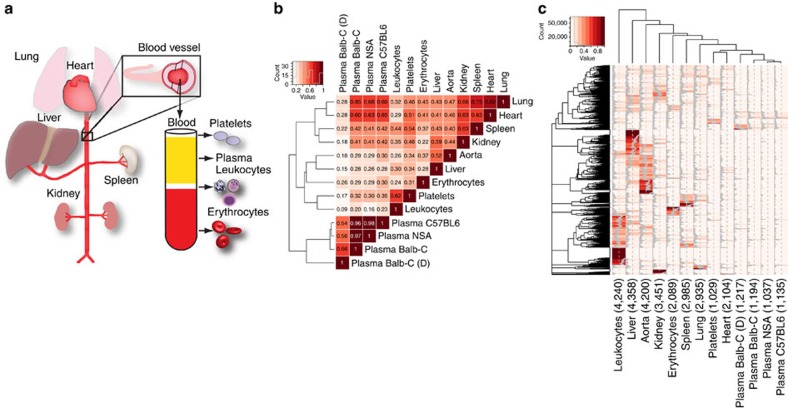
Construction of a tissue-specific protein abundance atlas. (**a**) Vascularized organs, plasma and cells adjacent to the blood plasma were collected from healthy Balb-C mice. The collected organs and cells were washed in PBS, homogenized, the proteins digested with trypsin and analysed by shotgun LC-MS/MS. (**b**) The scaled spectral counts from the LC-MS/MS analysis of the individual organs and cells were correlated using Pearson's r correlation coefficient, indicated by the numbers in the heat map. (**c**) Heat map of the scaled spectral counts of the different cell and organs. The grey lines in the heat map shows percentage of signal associated with a given organ or cell type. Total number of identified proteins per organ, blood vessel and cells is shown in brackets below the heat map.

Construction of a tissue atlas requires deep proteome analysis of isolated tissues and cells. In this study, we focused on heavily vascularized organs: liver, kidney, spleen, lung, heart, blood plasma and adjacent cell types such as leukocytes, erythrocytes, platelets and blood vessels (Fig. 1a). The organs, cell types and blood plasma were isolated from healthy Balb-C mice along with additional blood plasma samples from two different mouse strains: non-Swiss albino and C57BL6. Furthermore, for one additional Balb-C blood plasma sample, the most abundant plasma proteins were removed using a depletion column. The isolated organs and cells were homogenized and the extracted proteins were separated by SDS–polyacrylamide gel electrophoresis (SDS–PAGE). After in-gel digestion, peptides were analysed by shotgun liquid chromatography-tandem mass spectrometry (LC-MS/MS) analysis. The shotgun data were analysed using two databases, UniProt and PANTHER^8^; UniProt is larger and therefore contains more proteoforms compared with the smaller and more consistently annotated PANTHER database. At 1% false discovery rate, we identified 8,240 and 6,652 unique proteins, respectively, and this difference is a result of the more complex protein inference problem in the larger database as 95% of all identified peptides were shared between the two searches. The protein tissue distribution was established using percentage distribution of scaled spectral counts as previously described^9^,^10^. Comparing the distribution profiles between the proteomes using Pearson correlation revealed three major distinct groups (Fig. 1b). The most clearly defined sample group contains all the plasma samples and shows a high degree of similarity with a correlation of 0.96 or higher. The second group consists of proteomes from the organs: lung, liver, heart, spleen, aorta and kidney. The last group is the most distant to the other proteomes and includes the blood cells: platelets, leukocytes and erythrocytes. The protein distribution profiles are presented as a heat map in Fig. 1c (Supplementary Data 1). The heat map reveals that the number of unique proteins for a single cell type or organ is not evenly distributed. In general, there is a considerable overlap regarding protein content between the different organs and cells, but the protein abundance levels for a subset of the proteins are distinct between anatomical locations, as previously shown^11^,^12^,^13^.

### Enrichment of protein function in the protein tissue atlas

**Figure 2:**
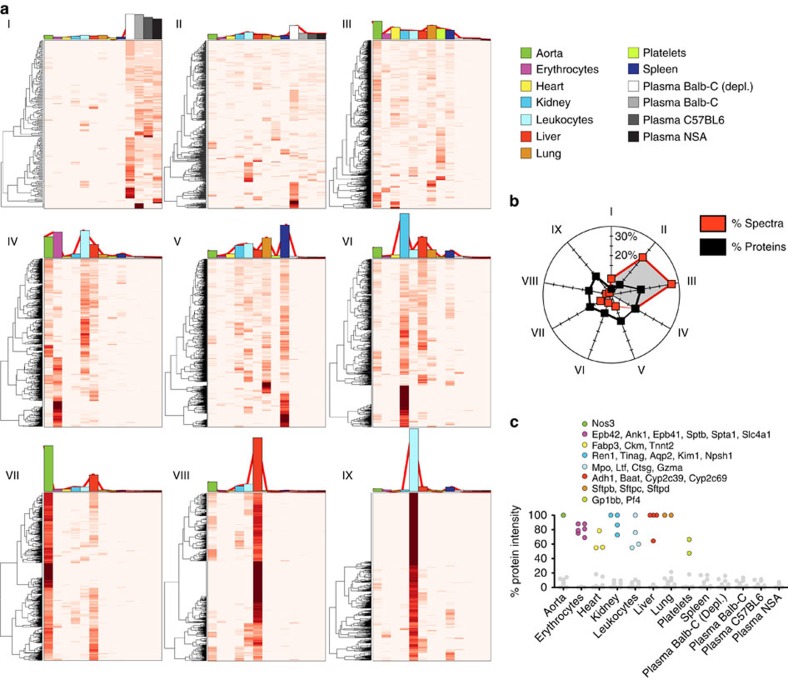
Protein function enrichment of the tissue-specific protein abundance atlas. (**a**,**b**) The quantitative protein profiles across the analysed organs and cells were subdivided into nine expression profiles using k-mean clustering and visualized as heat maps (I–IX). The average quantitative distribution of the proteins within one cluster is shown as coloured bar plots on top of the individual heat maps. The colours indicate organ, plasma or cell type. The total intensity of the proteins within the individual cluster was summed and total distribution between clusters are shown in **b**. (**c**) Examples of identified proteins with high tissue specificity, coloured according to the legend. The corresponding protein intensity in the other organs and cells are made grey for clarity. Data are based on scaled spectral counts from LC-MS/MS analysis.

To assess whether the tissue- and cell-derived proteome maps are enriched for expected protein functions, we subdivided all the identified proteins into nine clustered expression profiles using k-mean clustering. This was followed by functional enrichment analysis to determine functional groups associated with the different protein clusters. Data are presented as heat maps and the average spectral count distribution of the proteins within one cluster is shown as coloured bar plots of the individual heat maps. Two clusters contain proteins found in plasma (Fig. 2a I–II), whereas the other seven clusters include more tissue-specific proteins (Fig. 2a III–IX). For example, cluster I consists of proteins that are almost exclusively represented in plasma. This cluster contains the most typical plasma proteins and is as expected enriched for biological functions such as the complement and coagulation systems (Supplementary Data 2). Cluster II is distributed evenly across both plasma and tissue proteomes. Basic biological functions are enriched in this group such as protease inhibitors, cell–cell adhesion proteins and members of the host defense system. Several of the well-known plasma proteins also reside in this group, as the analysed organs are highly vascularized and contain residual amounts of blood plasma. The third cluster (III) consists of proteins that are present predominantly in all tissues and cells (Fig. 2a III) but hardly detectable in plasma. Many of the biological functions enriched in this group are essential for basal cellular function such as cytoskeletal proteins or proteins involved in intracellular signalling. A common feature for the first three groups (Fig. 2a I–III) is that they contain highly abundant proteins, contributing to over 64.5% of all measured MS spectra (Fig. 2b). Particularly, the plasma proteins that reside in cluster I and II are highly abundant. These two clusters contain 9.6% of all identified proteins but represents 33.2% of all MS spectra. The remaining clusters contain in contrast less abundant proteins and display to a larger extent more tissue-specific protein profiles (Fig. 2a IV–IX). These clusters reveal an enrichment of proteins with tissue-specific functions such as drug metabolism for liver (cytochrome P450 enzymes), contractile proteins for heart, proteins associated with surface tension in alveoli for lungs and ion transporters for kidney (Supplementary Data 1). Figure 2c highlights the tissue distribution of selected proteins with high tissue specificity (Supplementary Data 3). Interestingly, several of the proteins are detectable in blood plasma and some of them are, or have been, used in the clinical routine to determine, for example, myocardial infarction^14^ or to classify hypertension^15^. These results show that the protein distribution map can capture tissue-specific profiles correlating to the predominant biological functions associated with the analysed organs and cells.

### Tissue-specific protein profiles in healthy blood plasma

On the basis of the 130 separate shotgun LC-MS/MS injections, a total of 1,768 proteins were detectable in plasma from control animals. The tissue distribution of these 1,768 proteins across the analysed organs and cells can be used to infer the most likely origin. Here we used a 30% relative abundance across the tissues for a discrete protein tissue assignment. These tissue assignments have a considerable overlap with previously published proteome tissue maps^16^. We compared the results obtained in this work with a tissue-specific human data set based on RNA sequencing and immunohistochemistry data^16^. At a relative intensity threshold of 30% spectral abundance across the tissues, 100% of the true-positive matches could be recovered, corresponding to a false discovery rate of roughly 26% determined using a receiver operator characteristics (Supplementary Fig. 1). Furthermore, functional enrichment for the proteins in the subdivided groups reveals enrichment of tissue-specific functions (Supplementary Data 4).

**Figure 3:**
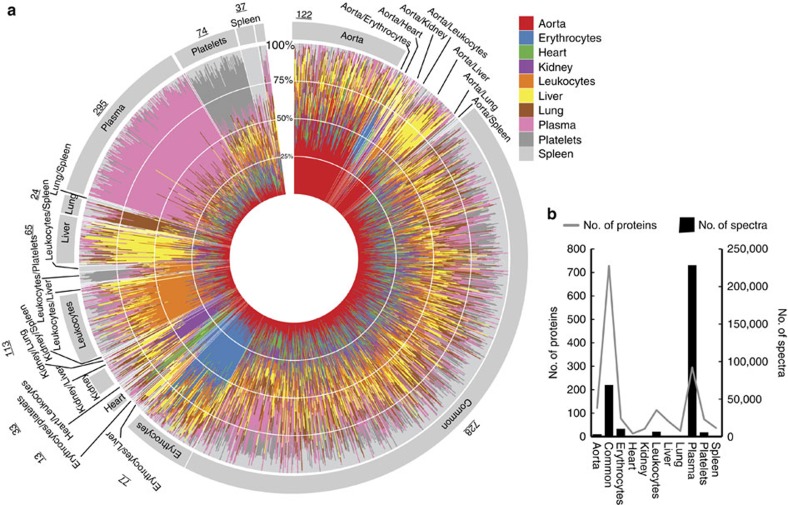
Tissue-specific protein in healthy blood plasma. The distribution of the protein intensity across the analysed organs and cells was determined by scaled spectral counts from LC-MS/MS analysis. (**a**,**b**) All the proteins detected in healthy plasma were plotted as individual bar plots in a circular polar histogram. The segments within the bar plots are coloured according to the colour scheme shown in the legend. The proteins were grouped according to similarity as shown by the colour arrangement in the polar histogram. The annotations outside the polar histogram indicate the predicted protein origin and the total number of proteins within one group. The number of identified proteins and the total intensity for the major groups identified in plasma are shown in **b**.

We visualized the protein distribution between tissues and cells for all the proteins detectable in blood plasma using a circular polar histogram^17^ (Fig. 3a). The polar histogram contains bar plots for all the 1,768 proteins arranged in a circular fashion and the relative distribution of protein levels across tissues and cells are indicated by colours. The graph captures the vast majority of known classical plasma proteins in the protein group denoted as ‘plasma' (pink colour in Fig. 3a), exemplified by proteins from the complement and coagulation systems. These proteins are relatively few in number but highly abundant in blood plasma accounting for close to 70% of the total MS spectra (Fig. 3b). This protein group includes typical plasma proteins discovered in Fig. 2a I–II. The remaining proteins (non-plasma protein) are predominantly present in the different organs or cells. The majority of these non-plasma proteins have an even distribution between organs and cells, denoted as ‘common' in Fig. 3a and Supplementary Figs 2 and 3. Together, these two protein groups (‘plasma' and ‘common') represent >90% of the detectable MS spectra in blood plasma from healthy animals (Fig. 3b). To summarize, these results show that the blood plasma proteome can be subdivided into two fractions. One smaller fraction primarily comprised highly expressed and well-characterized blood plasma proteins. The other fraction consists of protein predominantly expressed in the different organs and cells. This part is more numerous and contains proteins of low abundance, many of which are evenly distributed between the different organs and cells. Importantly, in the latter group several proteins reside with a high level of tissue specificity, primarily from cells present in the blood plasma. Finally, all the shotgun MS results were used to construct a spectral library as previously described^3^,^18^,^19^ representing one of the largest constructed spectral libraries for mice containing in total 66,857 unique peptides, corresponding to 258,027 spectra.

### Tissue-specific protein profiles in septic blood plasma

**Figure 4:**
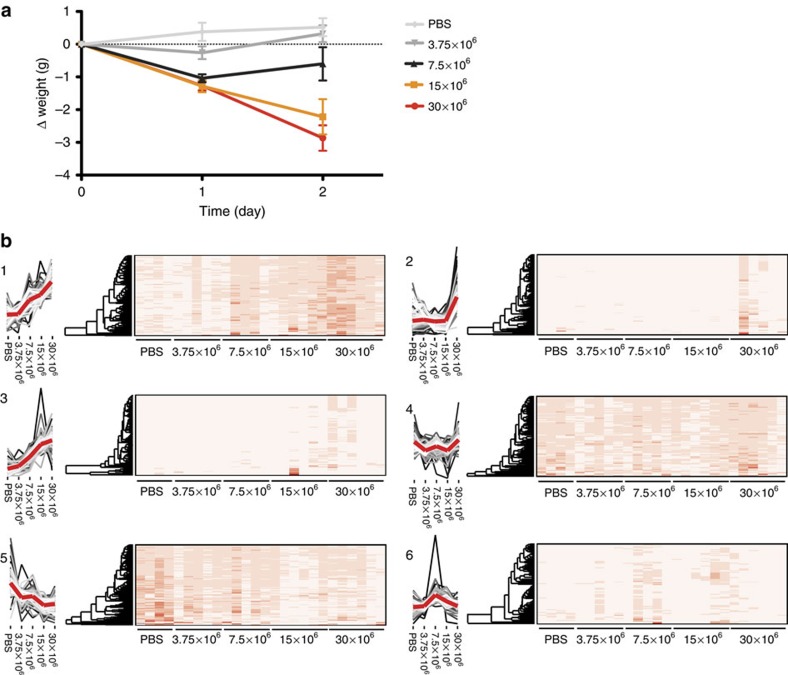
Quantitative changes of blood plasma proteins during severe Sepsis. In total, 26 animals were inoculated with *S. pyogenes* bacteria using different infectious doses (3.75 × 10^6^, 7.5 × 10^6^, 15 × 10^6^ and 30 × 10^6^) or PBS as control. The animals were killed after 48 h and citrated blood was collected using cardiac puncture. The blood plasma proteins were digested with trypsin followed by DIA-MS analysis. (**a**) Average weight loss of the animals within the dose groups of inoculated mice. (**b**) The proteins were clustered using t-SNE dimensionality reduction followed by PAM clustering and the abundance profiles for the six groups shown as line graphs in 1–6. The denser part of the graph is shown as increasing lighter coloured lines. The average intensity profile across the groups is shown as a thicker red line. The relative protein abundance for the individual animals are shown using heat maps.

The spectral library can be used to determine protein identity and quantity from raw data obtained via DIA-MS^4^ using previously published software solutions^3^,^18^. The tissue atlas provides the means to measure how surrounding tissues, all organs, blood vessel and cells, influence the composition of the blood plasma. We applied this strategy to determine changes in the plasma proteome in mice progressing from a non-septic to a septic status. To mimic the host response observed in sepsis, we inoculated wild-type Balb-C mice with a mouse virulent *Streptococcus pyogenes* strain by subcutaneous injection. In this experiment, we used 26 separate Balb-C mice in five different dose groups, where four mice served as control (PBS-injected). The remaining 22 mice were divided into four groups and subcutaneously infected with *S. pyogenes* bacteria with different concentrations (3.75 × 10^6^, 7.5 × 10^6^, 15 × 10^6^ and 30 × 10^6^). Animals were killed after 48 h and citrated blood was collected using cardiac puncture and 1 μl non-depleted plasma was used for DIA-MS analysis. The average weight loss within each group was directly correlated to the different bacterial concentrations demonstrating that the dose-response experiment mimics disease severity (Fig. 4a).

We were able to quantify 786 proteins using DIA-MS from <1 μl non-depleted plasma, increasing the number of identified proteins close to threefold compared with shotgun MS data using the same instrument and gradient length. Intriguingly the quantitative reproducibility was improved where more than 97.1% of the proteins were measured in 25 out of the 26 samples or more. The heat maps represent the protein expression of all the 786 identified plasma proteins in all the 26 animals subdivided into defined clusters using t-SNE^20^ dimensionality reduction followed by PAM clustering^21^ (Fig. 4b and Supplementary Data 5). Sepsis results in an extensive reorganization of the plasma proteome with distinct subgroups that were regulated differently. Clusters 1, 3 and 5 contain proteins that are induced or repressed in a dose-dependent manner while the proteins in cluster 4 and 6 remained relatively constant. The proteins in cluster 2 on the other hand displayed a distinct expression pattern and were primarily increased in some of the most critically ill animals. It appears that the concentration of these proteins suddenly increase once a certain threshold is reached, perhaps owing to cell necrosis and leakage of intracellular proteins. The different clusters suggest that there are two major response patterns in plasma during severe sepsis. One group of proteins appears to be mobilized against the invading pathogen relating to disease severity whereas the second group is altered as a consequence of the disease since they are primarily expressed in the most severely ill animals.

**Figure 5:**
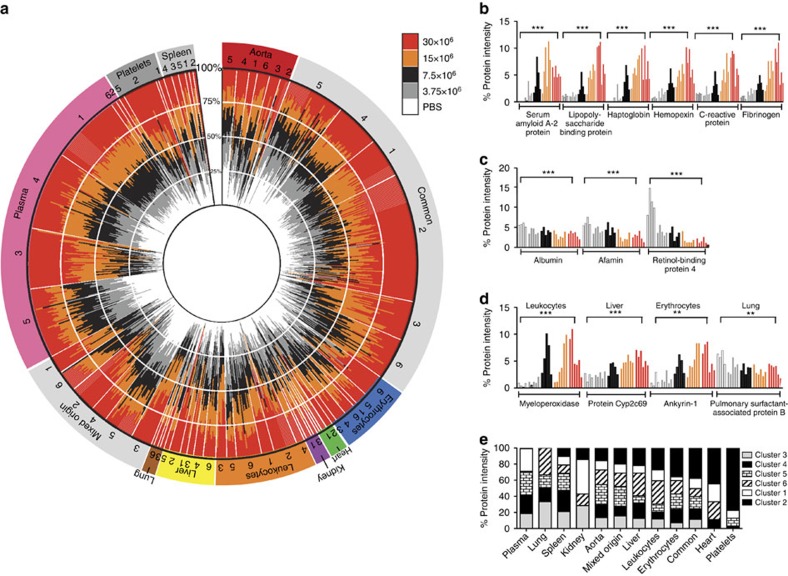
Sepsis results in increasing tissue-specific proteins in the blood plasma. The previously defined organ-, cell- and plasma-associated proteins identified in blood plasma using DIA was overlaid the t-SNE clusters. (**a**) Polar histogram illustrating quantitative protein changes of the defined tissue-, cell- and plasma-associated proteins across the five dose groups. The individual proteins are shown as bar plots and protein distribution coloured according to the dose group. The bar plots are shown in a circular polar histogram arranged in the same way as in Fig. 3a. The annotation outside the circle represents most likely organ, plasma and cell origin. The outer circle represents the proteins proposed primary localization defined by Fig. 3a and the clusters defined in Fig. 4b. The inner circle displays changes in protein abundance across the different dose groups. (**b**,**c**) Proteins increase or decrease in a dose-dependent fashion. (**d**) Examples of tissue proteins shown in Fig. 3c. (**e**) Total intensity of the organ, plasma and cell-specific associated proteins for every t-SNE clusters. Proteins were compared between untreated and the highest bacterial load and was evaluated by Student's *t*-test. The multiple-testing corrected (Hochberg) *P* values were calculated on the basis of a two-tailed distribution and unequal variance. *P<0.05, **P<0.01, ***P<0.001.

Adding the protein tissue atlas to the data matrix and visualizing the proteins in a polar histogram indicates how the specific tissue profiles are distributed over the clusters (Fig. 5a). The outer circle represents the primary localization of the proteins defined by Fig. 3a and the clusters defined in Fig. 4b. The inner circle depicts changes in protein levels across the different bacterial dose groups. For the plasma proteins, nearly 75% of the proteins are regulated in a dose-dependent manner (Fig. 5b,c), whereas 22% of the proteins remained constant (cluster 4). This information is supported by functional enrichment of cluster 1 and 3 where enriched groups are complement proteins, proteins associated with cell adhesion and the immune response and several positive acute-phase proteins (Supplementary Data 6). There are also several tissue-specific proteins that are regulated in a similar dose-dependent manner exemplified with some of the tissue-specific proteins shown in Fig. 3c (Fig. 5d). In contrast, several of the proteins originating from surrounding organs and cells display a protein expression pattern defined in cluster 2 (Fig. 4b). Interestingly, almost all (99%) of the proteins within this group (cluster 2 in Fig. 4b) are primarily derived from surrounding organs and cells including several known markers for cell necrosis such as lactate dehydrogenase (Supplementary Fig. 4)^22^. In addition, cluster 2 is enriched for functional protein groups essential for basal intracellular functions such as cell cycle, glycolysis and mitosis (Supplementary Data 6). Particularly proteins from platelets, erythrocytes, heart and leukocytes show a more drastic increase in the most affected animals (Fig. 5e). In general, several of these tissue-specific proteins are not regulated consistently in all animals within the same dose group indicating that the observed tissue-specific response varies across individual animals.

### Differential regulation of previously tested biomarkers

**Figure 6:**
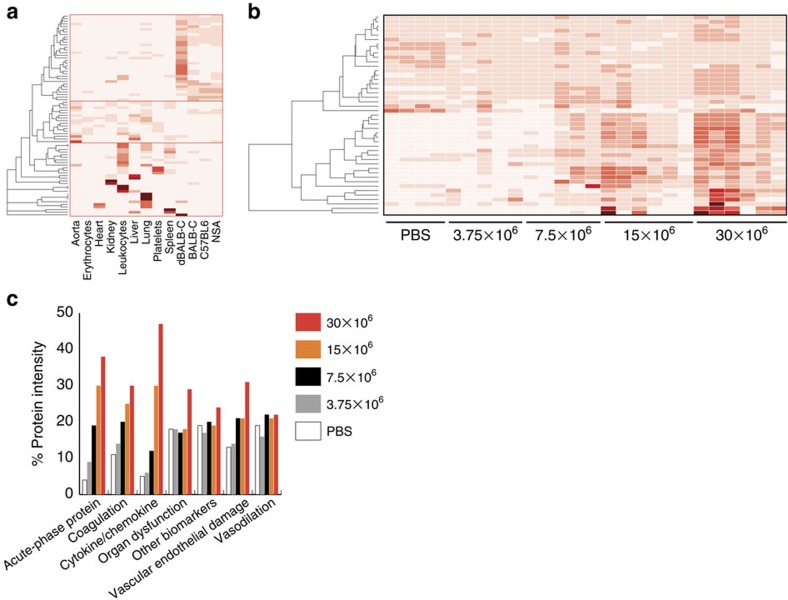
Abundance differences of known sepsis biomarkers. Previously tested human sepsis biomarkers^23^ were mapped to corresponding mouse orthologues identified in this study. (**a**) Heat-map indicating protein distribution of identified biomarkers across the analysed organs, cells and plasma based on scaled spectral counts from LC-MS/MS analysis. (**b**) Quantitative DIA-MS protein profiles of all identified biomarkers in blood plasma from the inoculated animals. Previous reports have grouped the putative biomarkers in annotated functional classes. The total intensity of the quantified proteins associated with specific annotated biomarker classes for the separate dose groups was summed up. (**c**) Total intensity quantitative changes for the annotated biomarker classes for the dose groups.

Several of the proteins identified in this study have been evaluated as biomarkers to predict sepsis disease progression in humans^23^. Previous reports have grouped the biomarkers into categories reflecting different sepsis-related characteristics such as acute phase proteins, coagulation, cytokines/chemokines, vascular endothelial damage, organ dysfunction and so on^23^. These human biomarkers were mapped to the mouse orthologous and the protein tissue atlas reveals that the predominant localization of these proteins are (i) plasma (ii) tissues and cells and (iii) highly tissue and cell specific (Fig. 6a). The majority of the biomarkers increase in protein concentration with increasing disease severity (Fig. 6b). By grouping the biomarkers according to biomarker class, we observe a dose-dependent increase of acute phase proteins, coagulation proteins and cytokines/chemokines (Fig. 6c). All of these groups are induced and are predominately localized to the blood plasma. There is also a smaller increase of biomarkers associated with vascular endothelial damage indicating that these animals have an increasing degree of vascular endothelial damage. For the biomarkers associated with organ dysfunction, there is only a noticeable increase in the highest infectious dose group. These results indicate that organ dysfunction is not a gradual process, but rather confined to some of the most infected animals. In contrast, there are only limited changes in the biomarker groups denoted as other biomarkers and vasodilation, respectively. These results demonstrate that the proposed strategy can measure several biomarker groups and quantitatively determine the level of change associated with several disease characteristics of sepsis. In addition, we anticipate that the tissue atlas can support in the future discovery of potentially new biomarkers in, for example, organ dysfunction.

## Discussion

Here we introduce an MS-based strategy to monitor the dynamics of tissue and cell-specific proteins in the blood plasma. The construction of a proteome-wide tissue atlas supports the inference of protein origin in the blood plasma. Subsequent analysis of blood plasma using DIA demonstrates how the spectral library can be used to quantitatively monitor close to 800 plasma- and tissue-specific proteins in a consistent and precise manner. We used this strategy to highlight how the surrounding tissue and cells influence the blood plasma in severe infectious diseases.

The blood plasma proteome has over the years been extensively analysed as a source for understanding diseases in the blood and for biomarker discovery studies. Several biomarker discovery studies aim at finding prognostic and diagnostic factors in the blood plasma for many different types of diseases. Still, the underlying factors behind the control of the blood plasma proteome, down to the individual protein level, remain to a large extent unclear. A recent report demonstrated that the blood plasma proteome among humans is variable and that genetic control and longitudinal variation affect protein levels and biological processes^24^ underlining the complex dynamics of proteins transitioning in and out of the blood plasma. The DIA-MS method is associated with quantitative reproducibility for a large number of proteins from the blood plasma, which can be used to facilitate the future assembly of large repositories of DIA-MS experiments. The tissue atlas spectral library described here can further advance our understanding of how surrounding tissues and cells influence the plasma proteome and how the plasma proteome is modified as a function of disease progression. This type of information is particularly relevant for systemic and multifactorial diseases such as sepsis. The quantification of biomarker groups associated with specific sepsis characteristics reveals how the groups have different regulation patterns as a consequence of disease severity. The variable response observed for several of the quantified biomarkers in this study may explain why several of the biomarkers have generated unsatisfying results when tested individually. Future large-scale DIA-MS experiments on blood plasma from animals with sepsis and human sepsis patients using the tissue atlas spectral library will more accurately enable the correlation between groups of biomarkers and disease progression, disease severity and disease outcome.

We predict that the strategy outlined here can be extended to measure how tissue proteins are regulated in many other pathological conditions improving our understanding of how surrounding tissues and cells can influence blood plasma as a consequence of disease. This information can be useful to increase our knowledge of how different pathophysiological processes distort the protein composition of healthy plasma to further explore the underlying pathophysiological mechanisms.

## Methods

### Bacteria

The *S. pyogenes* AP1 (40/58) strain of the M1 serotype was purchased from the World Health Organization Streptococcal Reference Laboratory in Prague, Czech Republic. S. *pyogenes* was grown overnight in Todd Hewitt broth supplemented with 0.5% yeast extract in the presence of 5% CO_2_ at 37 °C. An aliquot of these cells was added to fresh media and cultured to the exponential phase of growth (*D*_620_=0.4). The bacteria were washed twice with phosphate-buffered saline (PBS) and diluted to an appropriate concentration.

### Animal model

Female Balb-C mice were purchased from Janvier labs (Saint-Berthevin Cedex, France). The animals were housed under standard conditions of light and temperature and all the mice were fed laboratory chow and water *ad libitum*. Experiments were carried out with mice in the age group of 10 to 12 weeks. The animals were infected subcutaneously with *S. pyogenes* bacteria (3.75 × 10^6^, 7.5 × 10^6^, 15 × 10^6^ and 30 × 10^6^ in a total volume of 200 μl in the dorsal back), closely monitored for signs and symptoms of infection and weighed on a daily basis. Animals were killed using isofluorane until effect followed by cervical dislocation. Citrated blood was collected using cardiac puncture and mouse organs (heart, lung, liver, kidney, aorta and spleen) were harvested. The aorta (the ascending aorta down to the diaphragm) was dissected from 10 animals and pooled. Plasma from non-Swiss albino and C57BL6 mice were purchased from Innovative Research (Novi, MI, USA). The animal use protocols #M108-10, #M326-12 and #M327-12 were approved by the local Malmö/Lund Institutional Animal Care and Use Committee.

### Plasma preparation

Citrated blood was collected using cardiac puncture and centrifuged at 2,000*g* for 10 min and separate aliquots of the plasma supernatant were stored at 80 °C until analysis.

### Leukocyte isolation

Whole-pooled mouse blood (5 ml) from healthy control animals was layered on Polymorphprep (9 ml; Axis-Shield, Dundee, Scotland) and centrifuged at 700*g* for 60 min at 18°C. The leukocyte layer was thereafter recovered and suspended in 50 ml calcium- and magnesium-containing PBS. After centrifugation at 700*g* for 15 min, the remaining erythrocytes were removed by hypotonic lysis for 20 s. The cells were then pelleted at 250*g* (5 min), counted using a hemocytometer and resuspended in Na medium (containing 5.6 mM glucose, 127 mM NaCl, 10.8 mM KCl, 2.4 mM KH2PO4, 1.6 mM MgSO4, 10 mM Hepes and 1.8 mM CaCl2; pH adjusted to 7.3 with NaOH).

### Platelet and erythrocyte isolation

Whole-pooled mouse blood (5 ml) from healthy control animals was centrifuged at 200*g* for 10 min. The bottom part of the pelleted erythrocytes were recovered and suspended in PBS followed by a washing step (500*g*, 10 min). The erythrocyte pellet was thereafter resuspended in PBS. The platelet-rich plasma (PRP) was collected followed by a new centrifugation step at 2,000*g* for 10 min to pellet the platelets. The remaining plasma was removed and the platelets were resuspended in PBS.

### Organ preparation

Whole organs from one animal and cells from pooled animals were first washed in PBS followed by a homogenization step using a Polytron PT 2100 Homogenizer (Kinematica, Luzern, Switzerland). From each homogenized organ and cell, 250 μl samples were mixed with 100 mg 0.1 mm silica beads (Biospec Products, Bartlesville, OK, USA) and further homogenized using a FastPrep-96 instrument (1,600*g*, 180 s) (MP BIOMEDICALS, Santa Ana, CA, USA). The silicon beads were removed with a centrifugation step (14,000*g*, 1 min) and the protein concentration in the supernatant was determined with Pierce BCA Protein Assay Kit (Thermo Scientific, Waltham, MA, USA).

### SDS–PAGE analysis

Proteins from homogenized organs (100 μg) and 1 μl plasma was mixed with Laemmli sample buffer (Bio-Rad Laboratories Inc., Hercules, CA, USA) with 5% β-mercapto-ethanol (Sigma-Aldrich, St Louis, MO, USA) and run on precasted gels (Criterion 12+2 well comb, 45 ml, Bio-Rad Laboratories Inc). The gel was run at 60 V until the samples started to migrate and then the voltage was increased to 160 V. The gel was subsequently stained with GelCode Blue Stain Reagent (Thermo Scientific) for 30–60 min and excessive blue stain reagent was removed using deionized water.

### Depletion column

Highly abundant plasma proteins were removed with a Mouse 3 Multiple Affinity Removal Spin Cartridge as described by the manufacturer (Agilent Technologies, Santa Clara, CA, USA). Depleted plasma was concentrated using a Spin concentrator for proteins (5 kDa cut-off) according to the manufacturer's protocols (Agilent Technologies).

### In-gel digestion

Proteins from homogenized organs/cells (liver, kidney, spleen, lung, heart, blood vessel, leukocytes, erythrocytes and platelets) and 1 μl plasma were run on SDS–PAGE followed by in gel digestion^25^. The gel was cut and each lane was cut into 10 slices containing 100 mM ammonium bicarbonate (ABC, Sigma-Aldrich). To destain the GelCode Blue Stain Reagent from the gel pieces they were incubated with 50% acetonitrile (ACN, Sigma-Aldrich) 50 mM ABC to shrink and then reswelled with 100 mM ABC, this was repeated until no blue colour from the gel pieces could be detected. In the final washing step, the gel pieces were dehydrated using 100% ACN. The liquid was removed and the gel pieces were dried in a speedvac (miVac Duo concentrator, Genevac Ltd, Ipswich, UK) before the reduction and alkylation steps. The proteins were reduced with 100 μl 20 mM DL-Dithiothreitol (DTT) (Sigma-Aldrich) in 100 mM ABC for 60 min at 55 °C. The DTT were subsequently removed and proteins were alkylated with 200 μl 55 mM iodacetamide (IAA, Sigma-Aldrich) in 100 mM ABC for 45 min in the dark at room temperature, which was followed by several washing steps using 100 mM ABC and 100% ACN. Before each step the liquid was removed. The gel pieces were washed in 100 mM ABC followed by incubation in ACN. The gel pieces were then reswelled again by adding 100 mM ABC and finally dehydrated by adding ACN. The liquid phase was removed, and the gel pieces were completely dried in a speedvac. Proteins were digested by incubation with 100 μl trypsin (sequence grade modified trypsin porcine, Promega, Fitchburg, WI, USA; 10 ng μl^−1^) in 100 mM ABC overnight at 37 °C. Peptides were extracted by three changes of 5% formic acid in 50% ACN and by a final change of 100% ACN. The collected samples were concentrated in vacuum centrifuge and resuspended in 20 μl HPLC-water with 2% ACN, 0.2% formic acid. To all the samples, synthetic peptides (JPT Peptide Technologies, Berlin, Germany) were added for retention time calibration at the following concentrations: 1.0 pM GTFIIDPGGVIR; 5.5 pM TPVITGAPYEYR; 5.2 pM ADVTPADFSEWSK; 2.7 pM DGLDAASYYAPVR; 1.0 pM GAGSSEPVTGLDAK; 2.8 pM TPVISGGPYEYR; 2.9 pM GTFIIDPAAVIR; 3.4 pM YILAGVENSK; 2.7 pM VEATFGVDESNAK; 7.7 pM LGGNEQVTR. After in-gel digestion, peptides were analysed by shotgun LC-MS/MS analysis.

### In-solution digestion

One microlitre plasma from 26 separate Balb-C mice were digested and prepared for DIA-MS-analysis. Protein sample was dissolved in 8 M urea (Sigma-Aldrich) in 100 mM ABC, pH 8. The proteins were reduced with 5 mM Tris(2-carboxyethyl) phosphine (Sigma-Aldrich) for 60 min at 37 °C and alkylated with 10 mM IAA for 45 min in the dark at room temperature before diluting the sample with 100 mM ABC to a final urea concentration below 1.5 M. Proteins were digested by incubation with trypsin (1/100, w/w) overnight at 37 °C. Adding formic acid (Sigma-Aldrich), to pH 2–3 stopped the digestion. The peptides were cleaned up by C18 reversed-phase spin columns according to the manufacturer's instructions (Harvard Apparatus, Holliston, MA, USA). The collected samples were concentrated in vacuum centrifuge and resuspended in 50 μl HPLC-water with 2% ACN, 0.2% formic acid. To all the samples, synthetic peptides (JPT Peptide Technologies) were added as described earlier in the Methods section.

### LC-MS/MS analysis

All peptide analyses were performed on a Q Exactive Plus mass spectrometer (Thermo Scientific) connected to an EASY-nLC 1,000 ultra-high-performance liquid chromatography system (Thermo Scientific). For data-dependent acquisition, peptides were separated either on a PicoTip column (New Objective; ID 75 μm × 15 cm) packed with Reprosil-Pur C18-AQ 3 μm resin (Dr Maisch GmbH) or on an EASY-Spray column (Thermo Scientific; ID 75 μm × 25 cm, column temperature 45 °C). Column equilibration and sample load were performed using constant pressure at 250 (PicoTip) or 800 bar (EASY-Spray), respectively. A linear gradient from 5 to 35% acetonitrile in aqueous 0.1% formic acid was run for 120 min at a flow rate of 300 nl min^−1^. One full MS scan (resolution 70,000 @ 200 *m*/*z*; mass range 400–1,600 *m*/*z*) was followed by MS/MS scans (resolution 17,500 @ 200 *m*/*z*) of the 15 most abundant ion signals (TOP15). The precursor ions were isolated with 2 *m*/*z* isolation width and fragmented using higher-energy collisional-induced dissociation at a normalized collision energy of 30. Charge state screening was enabled, rejecting unassigned or singly charged ions. The dynamic exclusion window was set to 15 s and limited to 300 entries. The automatic gain control was set to 1e6 for both MS and MS/MS with ion accumulation times of 100 ms (MS) and 60 ms (MS/MS). The intensity threshold for precursor ion selection was 1.7e4.

For data-independent acquisition (DIA), peptides were separated using an EASY-Spray column (Thermo Scientific; ID 75 μm × 25 cm, column temperature 45 °C). Column equilibration and sample load was performed at 800 bar. A linear gradient from 5 to 35% acetonitrile in aqueous 0.1% formic acid was run for 120 min at a flow rate of 300 nl min^−1^. A full MS scan (resolution 70,000 @ 200 *m*/*z*; mass range from 400 to 1,200 *m*/*z*) was followed by 32 MS/MS full fragmentation scans (resolution 35,000 @ 200 *m*/*z*) using an isolation window of 26 *m*/*z* (including 0.5 *m*/*z* overlap between the previous and next window). The precursor ions within each isolation window were fragmented using higher-energy collisional-induced dissociation at a normalized collision energy of 30. The automatic gain control was set to 1e6 for both MS and MS/MS with ion accumulation times of 100 ms (MS) and 120 ms (MS/MS). The obtained raw files were converted to mzXML using the software tool ProteoWizard^26^.

### Mass spectrometry data analysis

Data were stored and managed using openBIS^27^ and all computational workflows were executed and managed using iportal^28^. Data integration, visualization and interpretation were done using DDB^29^,^30^,^31^. The shotgun data were analysed as described by Quandt *et al*.^32^; in short, X! Tandem (2013.06.15.1—LabKey, Insilicos, ISB)^33^ and OMSSA v.2.1.9 (ref. 34) was used to search the spectra against a protein reference database followed by peptideProphet^35^, iProphet^36^ and proteinProphet^37^, all part of the Trans-proteomic pipeline (TPP v4.7 POLAR VORTEX rev 0, Build 201403121010). The spectral library was created as described by Rosenberger *et al*.^19^; the spectral library was used by OpenSWATH to analyse the DIA-MS data. In short, each analyte (unique peptide sequence, charge state and post-translational modification profile) in the spectral library was used to extract ion chromatograms. The quantitative value was calculated by integrating the ion current for each of the fragments under the peak.

### Statistical analysis

Functional enrichments (part of the PANTHER database) for clusters and groups were computed using a simple bootstrapping algorithm^30^. Seventeen proteins were compared between untreated and the highest bacterial load and was evaluated by Student's *t*-test. The multiple-testing corrected (Hochberg) *P* values were calculated based on a two-tailed distribution and unequal variance. *P<0.05, **P<0.01, ***P<0.001.

## Additional information

**Accession codes**: The mass spectrometry proteomics data have been deposited to the ProteomeXchange Consortium42 via the PRIDE partner repository with the data set identifier PXD002896.

**How to cite this article:** Malmström, E. *et al*. Large-scale inference of protein tissue origin in gram-positive sepsis plasma using quantitative targeted proteomics. *Nat. Commun*. 7:10261 doi: 10.1038/ncomms10261 (2016).

## Supplementary Material

Supplementary Information Supplementary Figures 1-4 and Supplementary References

Supplementary Data 1 6652 proteins were identified after LC-MS/MS analysis at 1 % FDR when searching the PANTHER Mus musculus database. The table shows protein identification, uniprot accession number and spectral counts derived from respective organ and cell. The spectral count data is presented in a raw non-normalized format.

Supplementary Data 2 The tissue distribution of all the identified proteins was subdivided into nine clustered expression profiles using k-mean clustering. This was followed by functional enrichment analysis to determine functional groups associated with the different protein clusters. The table shows the enriched functional groups, the number of associated proteins, total number of proteins and z-score. Function groups with a z-score higher than 3.0 were included.

Supplementary Data 3 Selected proteins with previously determined to have high tissue specificity. The table shows, protein name, tissue annotation, uniprot accession number and scaled spectral counts.

Supplementary Data 4 The distribution of the protein intensity across the analyzed organs and cells was determined by scaled spectral counts from LC-MS/MS analysis. All 1768 proteins detected in healthy plasma were grouped based on their primary tissue localization as seen in Figure 3. This was followed by functional enrichment analysis to determine functional groups associated with the proteins primary localization. The table shows the enriched functional groups, the primary tissue localization, the number of associated proteins, total number of proteins and z-score. Function groups with a z-score higher than 3.0 were included.

Supplementary Data 5 26 Balb-C mice were subcutaneously infected with S. pyogenes bacteria with different concentrations (3.75x106, 7.5x106, 15x106 and 30x106) or with PBS (control). In total were 786 proteins identified and quantified using DIA-MS from one microliter non-depleted plasma. The table outlines protein name, uniprot accession number, PAM cluster, and intensity value determined with OpenSWATH.

Supplementary Data 6 786 identified plasma proteins using DIA-MS were subdivided into defined clusters using t-SNE dimensionality reduction followed by PAM clustering. This was followed by function enrichment analysis to determine functional groups associated with the different protein clusters. The table shows the enriched functional groups, the number of associated proteins, total number of proteins and z-score. Function groups with a z-score higher than 3.0 were included.
