# Supplementary material for: Large-scale inference of protein tissue origin in gram-positive sepsis plasma using quantitative targeted proteomics
Source: Nat Commun. 2016 Jan 6;7:10261. doi: 10.1038/ncomms10261 (PMC4729823; doi:10.1038/ncomms10261)
Supplement: Supplementary Information — Supplementary Figures 1-4 and Supplementary References [file ncomms10261-s1.pdf]

Supplementary Figure 1

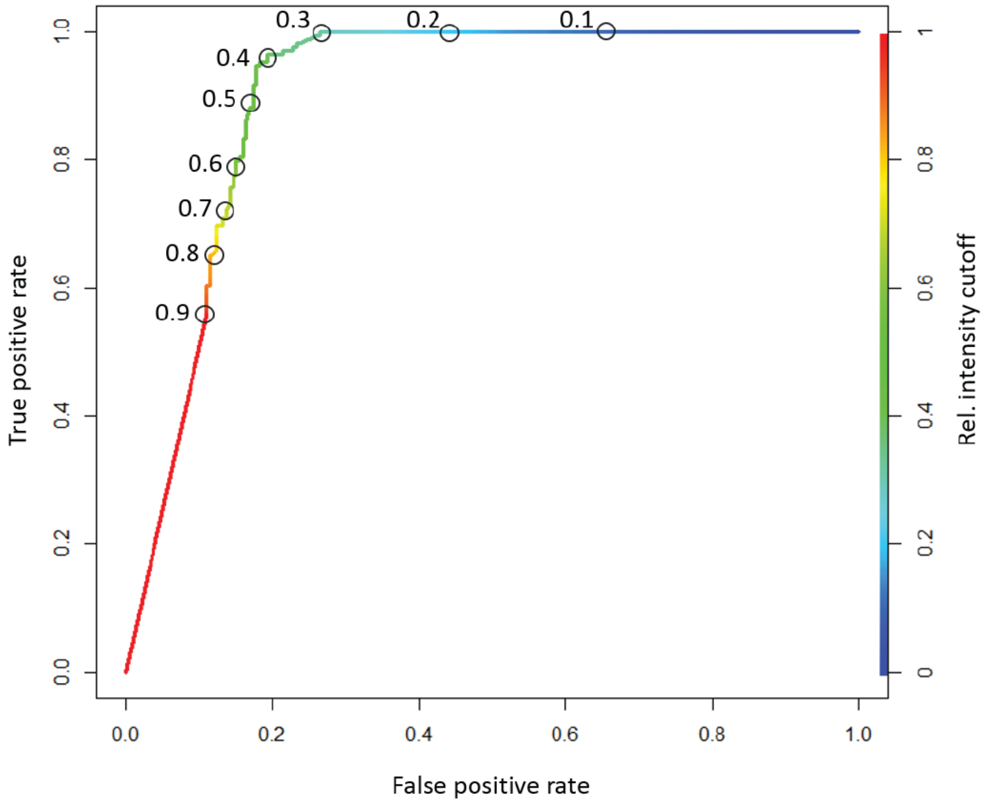

Supplementary Figure 1

Comparison between protein organ assignments from this study and Uhlén et al[1], in which the protein tissue distribution in human organs were analyzed by RNA sequencing and immunohistochemistry. The common organs between Uhlen et al and this study cover the lung, liver, kidney and heart. By mapping the murine proteins found in this study to their homologs in human and setting the Uhlén dataset as a reference for organ assignment, the two studies were compared using a binary classifier (same organ = TRUE; different organ = FALSE). The true positive rate was plotted against the false positive rate in a receiver operating characteristics (ROC) curve. At a relative intensity threshold of 30% spectral abundance across the tissues, 100% of the true positive matches could be recovered, corresponding to a false discovery rate of roughly 26%. Considering the large differences between the two studies, such as different organisms and homolog mapping, alternative analytical methods and technologies, and sample preparation, this result provides the rational for choosing the abundance cutoff for a discrete organ assignment at 30%. The ROC curve was created using the R package ROCR[2].

Supplementary Figure 2

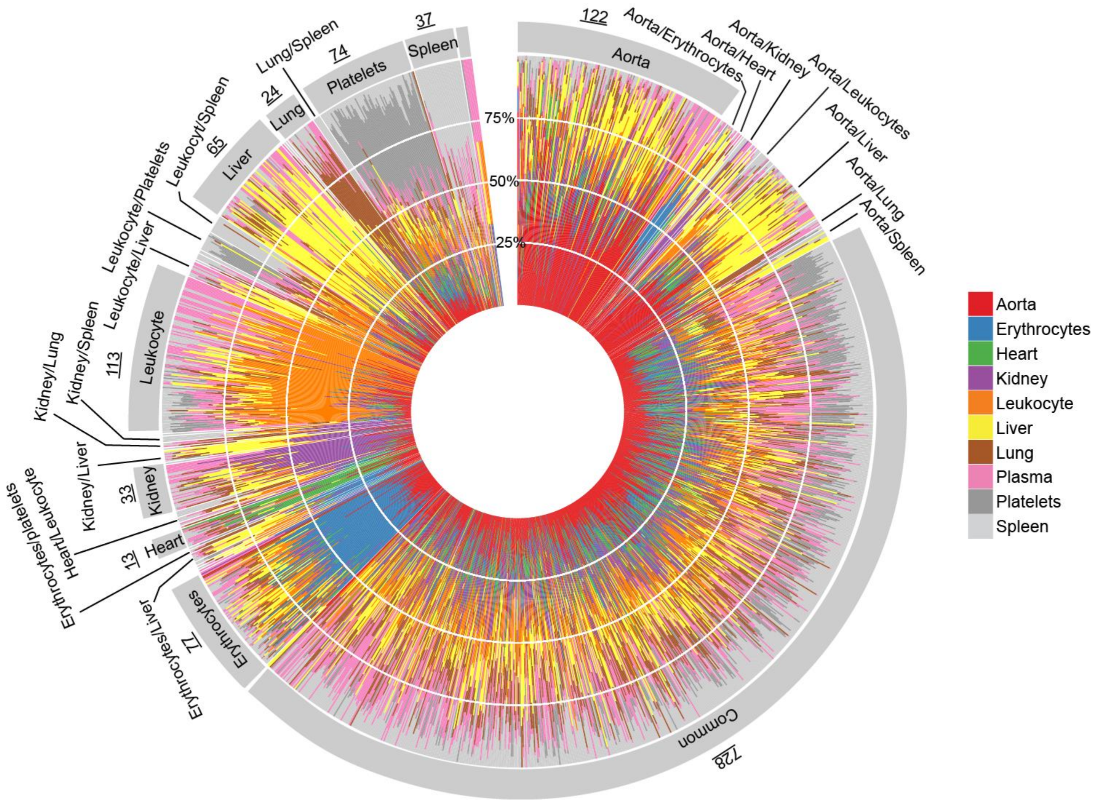

Supplementary Figure 2

The distribution of the protein intensity across the analyzed organs and cells was determined by scaled spectral counts from LC-MS/MS analysis. All 1768 proteins detected in healthy plasma were grouped based on their primary tissue localization as seen in Figure 3. Excluding the 295 proteins denoted as plasma proteins reveal the tissue distribution of 1468 tissue proteins detected in the blood plasma. These remaining 1468 proteins were plotted as individual bar plots in a circular polar histogram. The segments within the bar plots were colored according to the color scheme shown in the legend. The proteins were grouped according to similarity as shown by the color arrangement in the polar histogram. The annotations outside the polar histogram indicate the most likely protein origin and the total number of proteins within one group.

Supplementary Figure 3

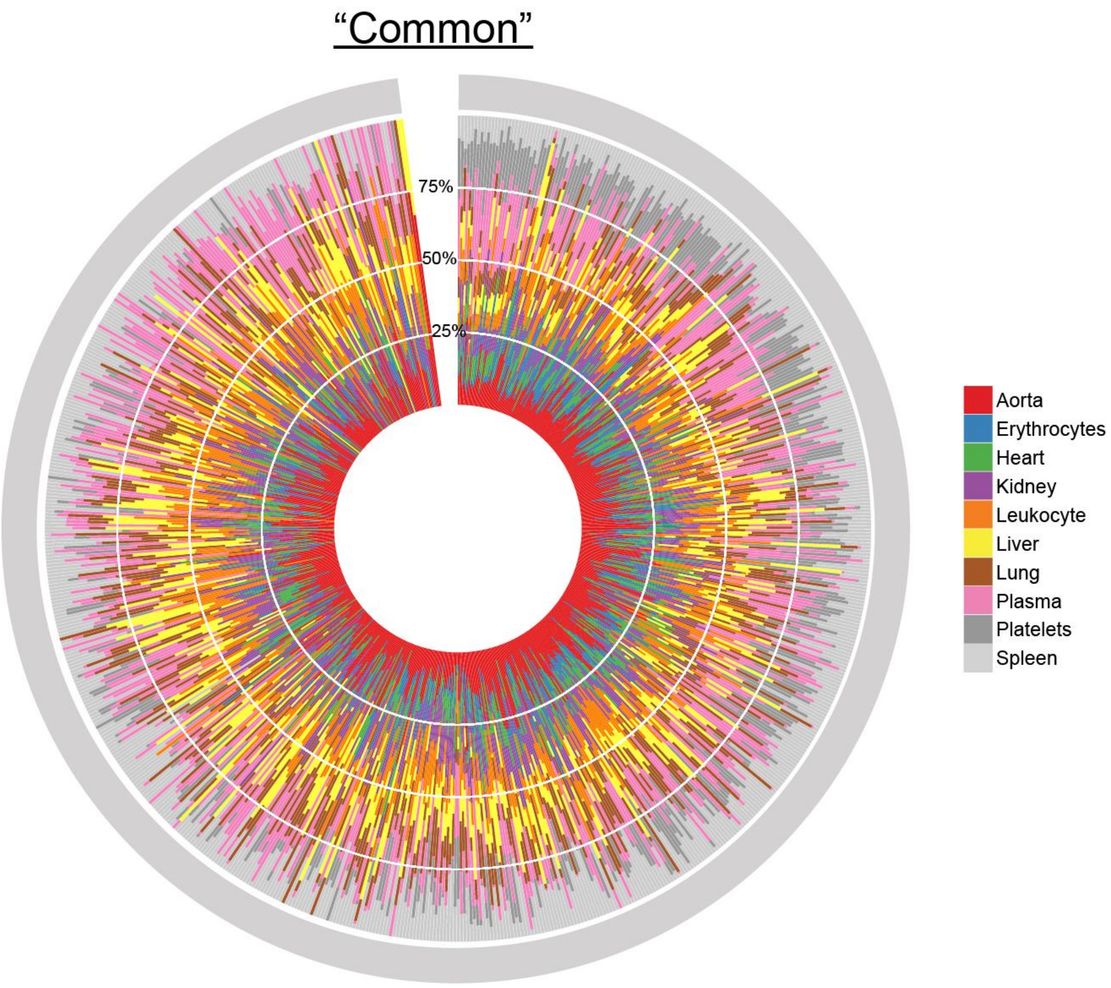

Supplementary Figure 3

The distribution of the protein intensity across the analyzed organs and cells was determined by scale spectral counts from LC-MS/MS analysis. The polar histogram visualizes the 728 detected in healthy plasma belonging to the group "common". Each protein was plotted as individual bar plots in a circular polar histogram and the segments within the bar plots were colored according to the color scheme shown in the legend. The proteins were grouped according to similarity as shown by the color arrangement in the polar histogram.

Supplementary Figure 4

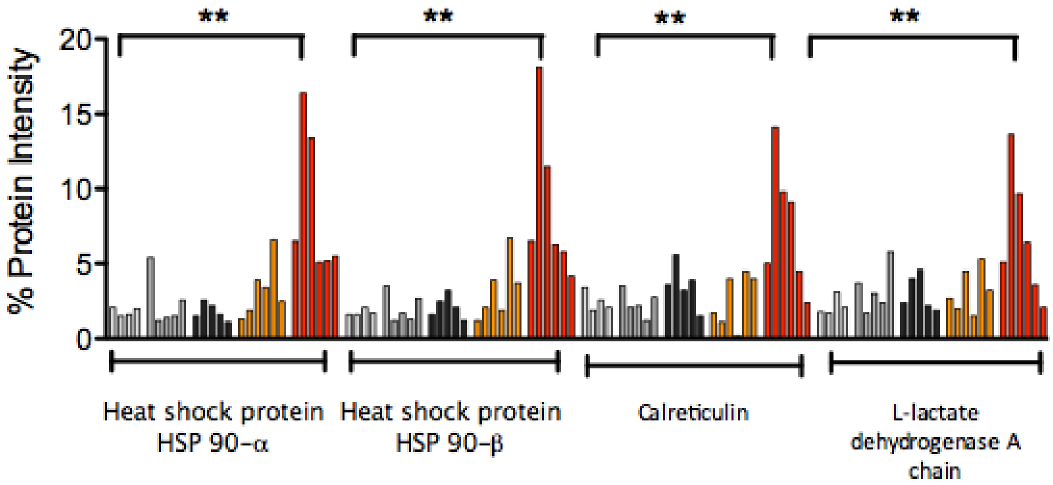

Supplementary Figure 4

In total 26 animals were inoculated with *S. pyogenes* bacteria using different infectious doses (3.75 x 10^6, 7.5 x 10^6, 15 x 10^6 and 30 x 10^6) or PBS as control. The animals were sacrificed after 48h and citrated blood was collected using cardiac puncture. The blood plasma proteins were digested with trypsin followed by DIA-MS analysis. The proteins were clustered using t-SNE dimensionality reduction followed by PAM clustering. The barplots indicate examples of known markers for cell necrosis associated to cluster 2 defined in Figure 4. Stars at the top of the graphs indicated p-value determined using student´s t-test between control and the highest dose group. ** = P<0.01

Supplementary References

1. Uhlen, M. *et al.* Tissue-based map of the human proteome. *Science* **347,** 1260419–1260419 (2015).
2. Sing, T., Sander, O., Beerenwinkel, N. & Lengauer, T. ROCR: visualizing classifier performance in R. *Bioinformatics* **21,** 3940–3941 (2005).
